# Associations between facet tropism and vertebral rotation in patients with degenerative lumbar disease

**DOI:** 10.1186/s40001-021-00622-7

**Published:** 2021-12-20

**Authors:** Yachao Ma, Peipei Huang, Zhipeng Tu, Zhou Yao, Zhe Wang, Zhuojing Luo, Xueyu Hu

**Affiliations:** Department of Orthopedics, Xijing Hospital, Air Force Medical University, No.127 Changle West Road, Xi’an, 710032 Shaanxi China

**Keywords:** Vertebral rotation, Zygapophyseal joint, Facet joint, Facet tropism

## Abstract

**Background:**

Vertebral rotation and facet tropism are very common in various lumbar degenerative diseases. Facet tropism means the presence of asymmetric angles on both sides of the facet joints. Studies have shown that facet tropism contributes to lumbar degenerative disease, and also inevitably leads to the asymmetry of movement and the imbalance of force, which may be possible to rotate the vertebral body. The aim of this study was to explore the correlation between lumbar vertebral rotation and facet tropism in patients with lumbar degenerative diseases.

**Methods:**

A total of 198 patients with lumbar degenerative diseases from 2018 to 2019 were enrolled. Five hundred and seventy vertebral rotation angles and 1140 facet angles were measured. The vertebral bodies are divided into non-rotation group (Group A) and rotation group (Group B) with the vertebral rotation angle of 3° as the boundary. The information including gender, age, BMI (body mass index), bone mineral density, history of smoking, drinking, hypertension, diabetes, diagnosis, segment distribution, and degree of facet degeneration were also counted. Using inter-class correlation coefficients (ICC) to test the reliability of measurement results. Univariate and multivariate logistic regression analysis were used to analyze the relationship between vertebral rotation and facet tropism.

**Results:**

The consistency of the ICC within the groups of the observers is above 0.8, with good agreement. The results of univariate analysis showed that facet tropism was significantly different between group A and group B (OR (odds ratio)  = 3.30, 95% CI  =  2.03–5.35, *P*  < 0.0001). Other significant factors were included as adjustment variables into the multivariate regression model. Three models were analyzed separately (Model 1: non-adjusted. Model 2: adjust for age; facet degeneration; Model 3: adjust for age; disease distribution; segment distribution; facet degeneration). The results showed that after adjusting the confounders, the correlation between facet tropism and vertebral rotation did not change (Model 1: OR  = 3.30, 95% CI  = 2.03–5.35, *P*  < 0.0001; Model 2: adjusted OR  = 2.87, 95% CI  = 1.66–4.97, *P*  = 0.0002, Model 3: adjusted OR  = 2.84, 95% CI  = 1.56–5.17, *P*  = 0.0006).

**Conclusion:**

Current research demonstrates that there is an association between vertebral rotation and facet tropism, suggesting that vertebral rotation may also have a certain degree of correlation with lumbar degenerative diseases.

## Background

Lumbar degenerative disease (LDD) is a series of diseases caused by degenerative changes in the lumbar spine [1]. It is a high-incidence disease among middle-aged and elderly people and seriously affects the quality of life [2, 3]. In recent years, with the increase of people’s life pressure and the aging process of the social population, the incidence of LDD is gradually increasing and showing a younger trend [4]. Among the many risk factors of LDD, facet tropism (FT) has been considered to be related to LDD such as low back pain, facet joint osteoarthritis, lumbar disc degeneration, recurrence of lumbar disc herniation, degenerative lumbar spondylolisthesis, and paravertebral muscle asymmetry [5–11]. FT means the asymmetric angles on both sides of the facet joints [11]. The feature of FT is that one facet is closer to the sagittal position than the other [7]. Although this is considered a normal feature of the thoracic spine, the more obvious asymmetry of the facet in the lumbar is considered to be the possible cause of the abnormal load pattern, which may eventually lead to pathological results [6, 12]. According to these studies, FT is an important risk factor for lumbar degenerative diseases.

Vertebral rotation (VR) is also a common phenomenon in LDD, especially in lumbar degenerative scoliosis. Studies have shown that the facet joints can limit the rotation of the vertebral body [13]. The asymmetry of the facet on both sides will inevitably lead to the asymmetry of movement and the imbalance of force [14], which may be possible to rotate the vertebral body. Similarly, the rotation of the vertebral body will also act on the facet joints on both sides. But there is no research on the relationship between VR and FT. The purpose of this study was to analyze the correlation between vertebral rotation and facet tropism. This study may provide some insight into whether vertebral rotation has an impact on lumbar degenerative diseases.

## Materials and methods

### Study population

The subjects were selected from patients with LDD in our hospital from 2018 to 2019. Inclusion criteria: 1. admission to the hospital for LDD, including disc herniation, spinal stenosis, degenerative spondylolisthesis; 2. with complete lumbar CT imaging data; 3. scanning plane parallel to the vertebral endplate; 4. no history of lumbar trauma or surgery. Exclusion criteria: 1. the degree of facet degeneration greater than III, which affects the measurement; 2. bone destruction such as fractures, tuberculosis or tumors; 3. vertebral deformities, lamina subfissure or other developmental abnormalities. According to related research [15], the vertebral bodies were divided into non-rotation group (Group A) and rotation group (Group B) with the boundary of 3° of vertebral rotation angle. The baseline information of the two groups of patients is shown in Table 1. The demographic information of each patient, including age, gender, and BMI (body mass index) are counted. The computed tomography (CT) image of the lumbar spine was used to measure the rotation angle and facet angle.

**Table 1:** Basic information of patients

	Group A	Group B	Standardized differences
Cases	311	259	
Age	50.3 ± 12.7	53.2 ± 12.1	0.23
Gender			0.02
Male [n (%)]	155 (49.84%)	126 (48.65%)	
Female [n (%)]	156 (50.16%)	133 (51.35%)	
BMI (kg/m^2^)	24.2 ± 3.2	24.07 ± 3.0	0.04
Disease distribution			0.36
Lumbar disc herniation [n (%)]	251 (80.71%)	197 (76.06%)	
Lumbar spinal stenosis [n (%)]	33 (10.61%)	54 (20.85%)	
Lumbar degenerative spondylolisthesis [n (%)]	27 (8.68%)	8 (3.09%)	
Segment distribution			0.28
L1/2 [n (%)]	22 (7.07%)	30 (11.58%)	
L2/3 [n (%)]	68 (21.86%)	63 (24.32%)	
L3/4 [n (%)]	68 (21.86%)	46 (17.76%)	
L4/5 [n (%)]	92 (29.58%)	54 (20.86%)	
L5/S1 [n (%)]	61 (19.61%)	66 (25.48%)	

### Measurements

**Fig. 1:**
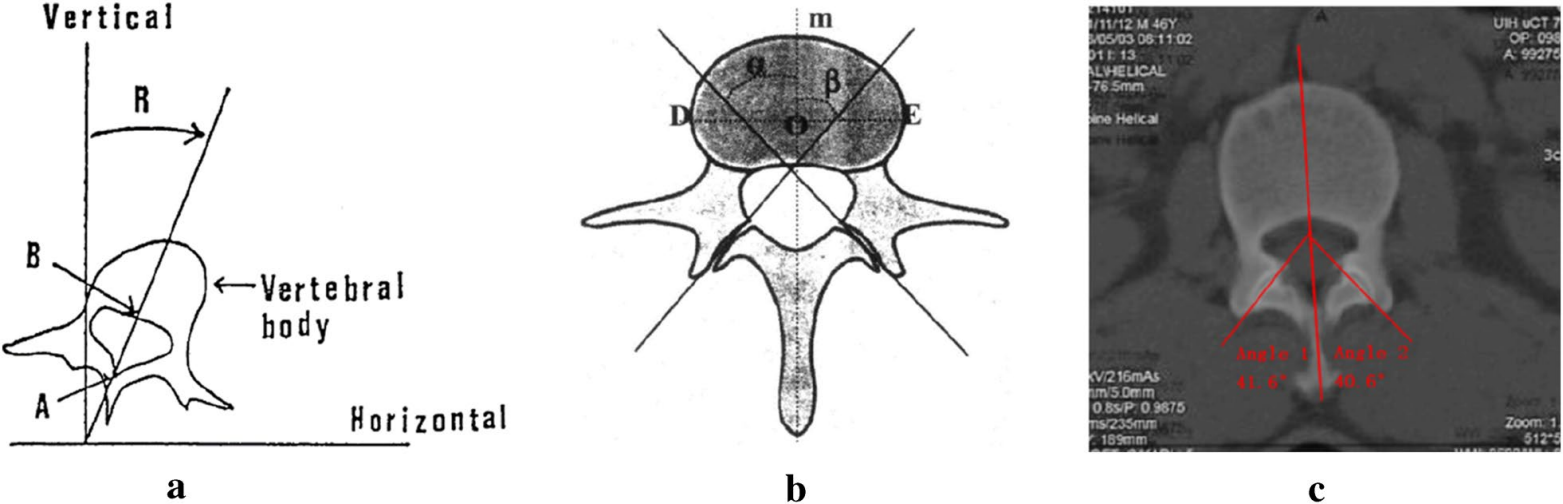
**a** The rotation angle is the angle between the midline of the vertebral body and the sagittal line. **b** The angle subtended by the plane of the facet with the sagittal plane in the midline of the vertebral body is the facet angle. **c** Diagram shows the measurement of rotation angle of L2 vertebral body and facet angle of L1/2 segment

**Fig. 2:**
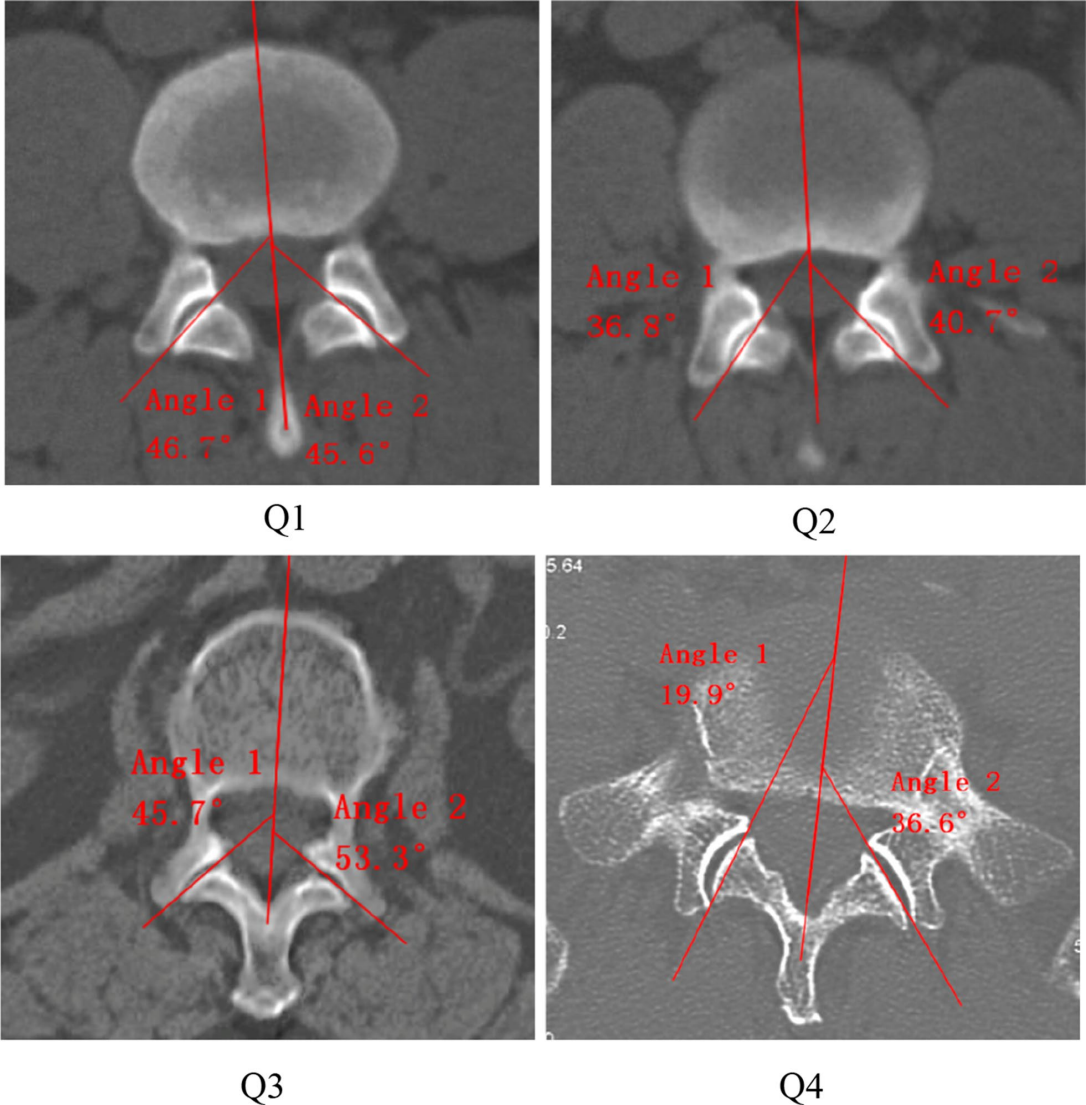
Examples of the four groups of facet angle differences

CT can show bone structure more clearly and provide accurate anatomical location [16]. Therefore, the measurement was done on the CT image. The images were randomly mixed with hidden patient information and numbered to ensure independent evaluation of the data by the observers using a blind method. Three experienced spine surgeons underwent one hour training before the start of the study. Each data was measured twice by three surgeons separately with an interval of 4 weeks to evaluate the intra- and inter-observer agreement, ensuring the accuracy of data acquired. The upper endplate layer of the vertebral body was selected for the layer of measurement. Because this level can better display the facet joint and reduce errors [17]. The Aaro-Dahlborn method [18] was used to measure the rotation angle of the vertebral body (Fig. 1a). Facet angle is the angle subtended by the plane of the facet with the sagittal plane in the midline of the vertebral body (Fig. 1b). The differences of facet angles were divided into four groups for analysis based on the quartile:  < 2.3° (Q1), 2.3°–4.6° (Q2), 4.6°–8.5° (Q3),  ≥ 8.5° (Q4) (Fig. 2). Facet degeneration is divided into 4 grades according to the standard of Patllria et al. [19]: I normal, II mild degeneration, III moderate degeneration, IV severe degeneration (Table 2). Surgimap 2.3 (Nemaris Inc., New York, NY, USA) was used for measuring.

**Table 2:** Grading of facet joint osteoarthritis (FJO)

Degeneration degree	Standard
I (normal)	Normal facet joint (joint gap is 2–4 mm)
II (mild)	Narrow space (< 2 mm) and/or mild osteophytes and/or mild hypertrophy of facet joints
III (moderate)	Narrow space (< 2 mm) and/or moderate osteophytes and/or moderate hypertrophy of facet joints and/or mild erosion of subarticular cartilage
IV (severe)	Narrow space (< 2 mm) and/or large osteophytes and/or severe hypertrophy of facet joints and/or severe erosion of subarticular cartilage and/or subchondral cysts

### Statistical analyses

The inter-class correlation coefficients (ICC) were calculated for the evaluation of the inter-observer reliability and intra-observer reproducibility. ICC greater than 0.9 indicates good consistency. Variables with *p* value <  0.20 were included in the multivariable logistic regression for controlling the possible effect of confounders. *P*  < 0.05 is considered statistically significant. Empower Stats and R software (R version 3.6.1) was used for statistical analysis.

## Results

A total of 570 vertebral rotation angles and 1140 facet angles from 198 patients were included and measured. There are 311 vertebral bodies and 622 facets in Group A (49.8% were from males and 50.2% were from females). 259 vertebral bodies and 518 facets in Group B (48.7% were from males and 51.3% were from females) (Table 1). ICC between and within the observers were above 0.9, with good consistency, demonstrating the reliability of the measurement results and the repeatability of the measurement method.

In this study, basic information including age, gender, BMI, bone mineral density, drinking, smoking, diabetes, hypertension, segmental distribution, diseases distribution, and grading of facet degeneration were included in the univariate analysis. The results showed that facet tropism was significantly related to vertebral rotation (OR  = 3.30, 95% CI  = 2.03, 5.35, *P*  < 0.0001). There were also differences in age, facet degeneration, segment distribution, and disease distribution between the two groups (Table 3).

**Table 3:** Univariate regression analysis of vertebral rotation

Factors		OR (95% CI)	*P* value
Age	51.66 ± 12.54	1.02 (1.01, 1.03)	0.0069
Gender			
Male [n (%)]	281 (49.30%)	Reference	
Female [n (%)]	289 (50.70%)	1.05 (0.75, 1.46)	0.7771
BMI	24.13 ± 3.10	0.99 (0.93, 1.04)	0.6699
Smoking			
No [n (%)]	458 (80.35%)	Reference	
Yes [n (%)]	112 (19.65%)	1.26 (0.83, 1.90)	0.2800
Drinking			
No [n (%)]	537 (94.21%)	Reference	
Yes [n (%)]	33 (5.79%)	0.88 (0.43, 1.79)	0.7203
Diabetes			
No [n (%)]	550 (96.49%)	Reference	
Yes [n (%)]	20 (3.51%)	1.21 (0.50, 2.95)	0.6770
Hypertension			
No [n (%)]	457 (80.18%)	Reference	
Yes [n (%)]	113 (19.82%)	0.90 (0.59, 1.36)	0.6207
Bone mineral density	− 1.14 ± 1.63	1.03 (0.90, 1.18)	0.7094
Disease distribution			
Lumbar disc herniation [n (%)]	87 (15.26%)	Reference	
Lumbar spinal stenosis [n (%)]	448 (78.60%)	0.48 (0.30, 0.77)	0.0023
Lumbar degenerative spondylolisthesis [n (%)]	35 (6.14%)	0.18 (0.07, 0.45)	0.0002
Segment distribution			
L1/2 [n (%)]	52 (9.12%)	Reference	
L2/3 [n (%)]	131 (22.98%)	0.68 (0.36, 1.30)	0.2425
L3/4 [n (%)]	114 (20.00%)	0.50 (0.26, 0.96)	0.0389
L4/5 [n (%)]	146 (25.61%)	0.43 (0.23, 0.82)	0.0104
L5/S1 [n (%)]	127 (22.28%)	0.79 (0.41, 1.52)	0.4861
Grading of facet degeneration			
I	418 (73.44%)	Reference	
II	117 (20.50%)	2.40 (1.57, 3.67)	< 0.0001
III	35 (6.06%)	2.20 (1.08, 4.49)	0.0293
Facet asymmetry			
Q1	139 (24.39%)	Reference	
Q2	145 (25.44%)	1.00 (0.61, 1.63)	0.993
Q3	138 (24.21%)	1.79 (1.10, 2.90)	0.0184
Q4	148 (25.96%)	3.30 (2.03, 5.35)	< 0.0001

**Fig. 3:**
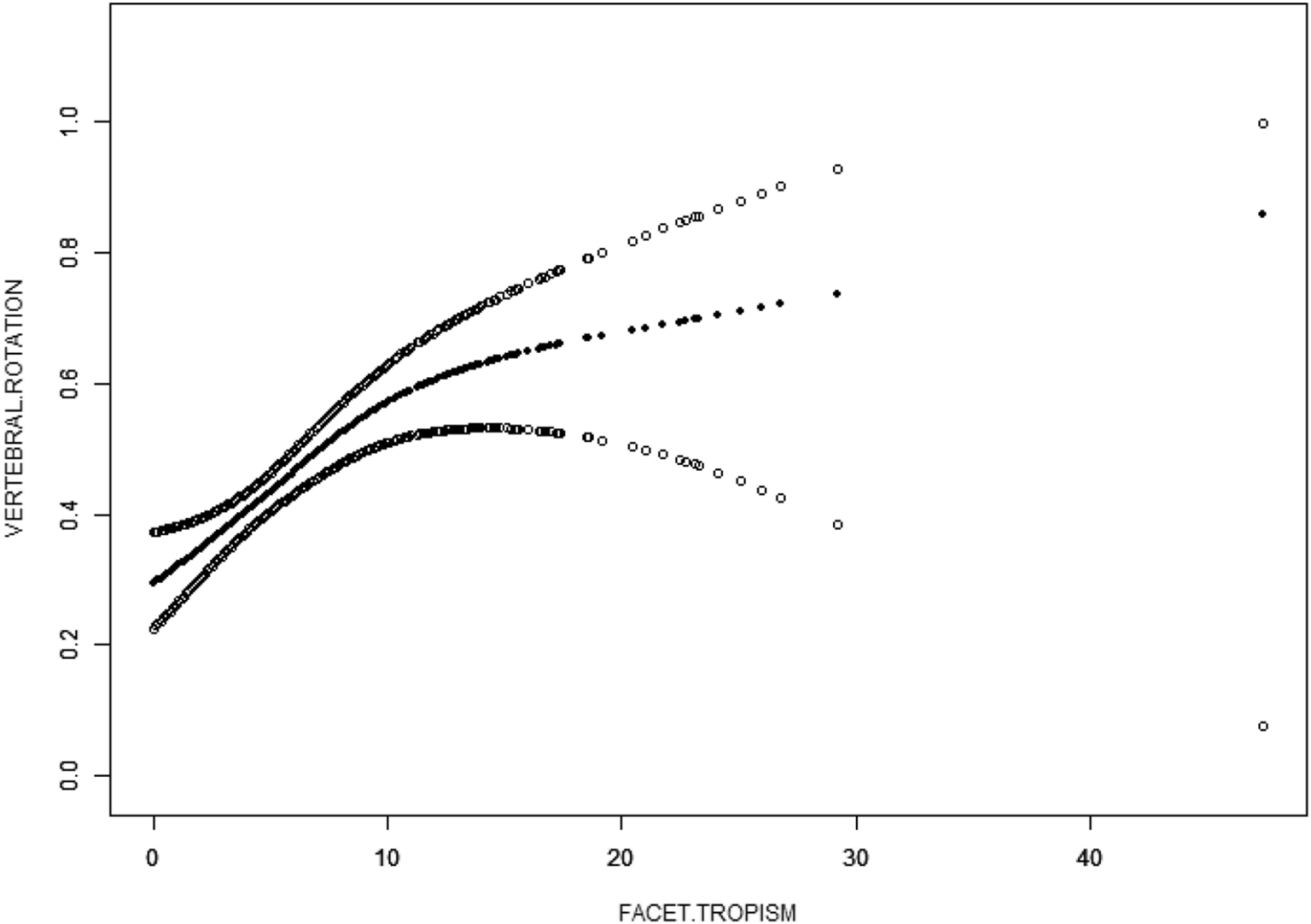
The relationship between vertebral rotation and facet tropism. The middle solid arc represents the smooth fitting curve between the variables. The dotted lines on both sides represent the 95% confidence interval of the fitted curve. Adjusted for age, facet degeneration grade, segment distribution and disease distribution

According to the results of univariate regression analysis, we focused on the influence of facet tropism on vertebral rotation, and the remaining factors with *P* value  < 0.20 were included as adjustment variables into the multivariate regression model. Three models were established (Model 1: did not adjust for any confounding factors, Model 2: adjusted age and facet degeneration, and Model 3: further adjusted segment and disease distribution). The results showed that after adjusting for the confounders, the correlation between facet tropism and vertebral rotation did not change (Model 1: OR (95% CI)  = 3.30 (2.03, 5.35), *P*  < 0.0001, Model 2: adjusted OR (95% CI)  = 2.87 (1.66, 4.97), *P*  = 0.0002, Model 3: adjusted OR (95% CI)  = 2.84 (1.56, 5.17), *P*  = 0.0006). As the degree of asymmetry increases, the ratio of vertebral rotation also increases significantly (Table 4). In the generalized additive model (GAM), a nonlinear relationship between vertebral rotation and the degree of facet tropism was found (Fig. 3).

**Table 4:** Multivariate regression analysis

Facet tropism	Model 1		Model 2		Model 3	
	OR (95% CI)	*P* Value	OR (95% CI)	*P* Value	OR (95% CI)	*P* Value
Q1	Reference		Reference		Reference	
Q2	1.00 (0.61, 1.63)	0.9930	0.96 (0.58, 1.58)	0.8788	0.92 (0.55, 1.55)	0.7502
Q3	1.79 (1.10, 2.90)	0.0184	1.63 (0.99, 2.68)	0.0533	1.58 (0.94, 2.69)	0.0871
Q4	3.30 (2.03, 5.35)	< 0.0001	2.87 (1.66, 4.97)	0.0002	2.84 (1.56, 5.17)	0.0006

Model 1: non-adjusted; Model 2: adjust for age; facet degeneration; Model 3: adjust for age; disease distribution; segment distribution; facet degeneration

## Discussion

The facet joint is a synovial joint surrounded by cartilage and joint capsule. The bilateral facet joints and intervertebral disc constitute three-joint complex, which are the basic movement unit of the lumbar spine. Although normal discs allow movement in all planes, facet joints are naturally limited by their geometric structure and cystic attachment. The facet joints have shown a progressively more coronal orientation from the proximal end to the distal [20]. The orientation of the lumbar facet joints allows a larger range of flexion movements, but at the same time can prevent large rotational instabilities. When the vertebral body tries to rotate axially, the adjacent facet can effectively lock together [21]. This mechanism is believed to protect the vertebral body from excessive rotation and maintain stability [22].

According to the anatomical relationship, the rotation of the vertebral body inevitably acts on the facet on both sides, causing changes in stress. The finite element analysis conducted by Zeng et al. [23] showed that the rotation range of the vertebral body increased after the facet joint was removed, which clearly indicated that the facet joint has the effect of restricting rotation. Zheng et al. [24] simulated the flexion, extension, lateral curvature and rotation of the spine in a finite element analysis, and found that the stress concentration of facet joint was the most obvious in the case of rotation. These studies show that there is an interaction between the facet and vertebral body.

Facet tropism is a common phenomenon in the spine, which means that the angles of the facet on both sides are different. Mohanty et al. [20] analyzed the facet angles of 566 normal vertebral bodies. The incidence of facet tropism ranged from 22.42 to 47.82%. Biomechanics studies have increased the understanding of the face tropism. Kim et al. [25] conducted a finite element model analysis of 3 different facet orientations (50°, 55° and 60° relative to the coronal plane) and 1 facet tropism (50° on the right side and 60° on the left). The results showed that there is no difference in the pressure gradient of the intervertebral disc between the different facet orientation models. However, in the facet tropism model, the pressure on the disc and facet joint increased the most, indicating that the facet tropism is more susceptible to external forces or shear forces. And the asymmetry of the facet will cause the vertebral body to receive greater shearing force during the rotation process. But the relationship between vertebral rotation and facet tropism has not been studied yet. In this study, by measuring 570 vertebral rotation angles and 1140 facet angles, we found a correlation between them. This result is in line with the anatomical characteristics of the vertebral body and previous related studies. But the causal relationship between them needs to be confirmed by long-term clinical studies, and maybe they influence and promote each other.

Masharawi et al. [26] analyzed 4080 vertebrae from T1 to L5 in 240 normal adults, and concluded that the asymmetry of the thoracic facet joints is normal, but it has a certain correlation with the pathological changes in the lumbar spine. Weinberg et al. [27] obtained 599 cadaveric lumbar spines from the Hamann–Todd osteological collection. They found that the average facet tropism increased from rostrally to caudally from 6.1 ± 5.5 degrees at T12–L1 to 11.2 ± 8.6 degrees at L5–S1. And they suggested that facet joints do possess the ability to remodel over time, perhaps in response to perturbations of sagittal balance, osteophyte formation, or other yet to be determined factors. Up to now, there are many reports of facet tropism in the study of disc herniation, degenerative spondylolisthesis, low back pain, ligamentum flavum thickness and other lumbar degenerative diseases [7, 10, 20, 21, 28–33]. Therefore, vertebral rotation may also be related to degenerative diseases of the lumbar spine to a certain extent.

This study has some limitations. First, according to research, the observation of the facet should be done within a period of time, not at a point in time, because the facet joints will change over time [14, 27]. In addition, different grading standards for vertebral rotation and facet tropism may have different results.

## Conclusions

Current research demonstrates that there is an association between vertebral rotation and facet tropism. This suggests that the segments of rotated vertebral body may have a higher risk of degeneration. And long-term prospective studies need to be conducted to study the causality between them.

## Data Availability

The datasets used and/or analyzed during the current study are available from the corresponding author on reasonable request.
